# Viscotoxin and lectin content in foliage and fruit of *Viscum album* L. on the main host trees of Hyrcanian forests

**DOI:** 10.1038/s41598-022-14504-3

**Published:** 2022-06-20

**Authors:** Sanaz Yousefvand, Farnoosh Fattahi, Seyed Mohsen Hosseini, Konrad Urech, Gerhard Schaller

**Affiliations:** 1 Faculty of Natural Resources and Marine Sciences, Tarbiat Modares University, Noor, Iran; 2 Department of Forest Science and Engineering, Faculty of Natural Resources and Marine Sciences, Tarbiat Modares University, Noor, Iran; 3 Hiscia Research Institute, Society for Cancer Research, Arlesheim, Switzerland

**Keywords:** Biochemistry, Cancer, Chemical biology, Ecology, Physiology, Plant sciences

## Abstract

Mistletoe (*Viscum album* L.) is a hemiparasitic plant that absorbs water and nutrients from the host tree. Mistletoe contains two groups of cytotoxic, immunomodulatory and antitumor proteins, viscotoxins and lectins. This study evaluated the quantity and quality of viscotoxins and total lectins in the stems with leaves (foliage) and fruit of mistletoe on *Parrotia persica* and *Carpinus betulus* in September with immature green berries and in December with mature white berries. *Viscum album* L. plants were harvested from host species located in the Hyrcanian forests of Iran in 2019. The highest level of viscotoxins was detected in the December foliage of *V. album* settled on *C. betulus* (9.25 mg/g dry weight [DW]), and the highest content of lectins was found in the December foliage of *V. album* settled on *P. persic*a (0.79 mg/g DW) and *C. betulus* (0.73 mg/g DW) respectively. The immature green berries of *V. album* from both host species contained much higher concentrations of viscotoxins and lectins than the mature white berries. Four isoforms of viscotoxins, viscotoxin A1, A2, A3 and B could be identified in all samples of both host species. Viscotoxin A3 was the predominant viscotoxin isoform followed by viscotoxin A1.

## Introduction

The use of herbal medicines is very popular. The World Health Organization estimates that up to 80% of people rely on traditional remedies such as plant bioactive compounds^1,2^.

Cancer is one of the most prevalent reasons for death and indeed second in the cause of death just after cardiovascular disease worldwide^3,4^. Since common treatments such as chemotherapy are connected with adverse drug effects, well-tolerated promising treatment options are expected to result from research on natural products derived from plants. More than 60% of confirmed drugs for cancer therapy are derived from medicinal plants^5–7^.

Mistletoe (*Viscum album* L.) from the Santalaceae family is an evergreen, perennial, hemiparasitic plant that absorbs water and nutrients from the host tree through a root-like organ called haustorium^8–11^. European mistletoe is divided into four subspecies with different host trees: *V. album* subsp. *album* L. on deciduous trees, *V. album* subsp. *abietis* (Wiesb.) only on firs (*Abies* spp.), *V. album* subsp. *austriacum* (Wiesb.) on pines (*Pinus* spp.), very rarely spruce (*Picea abies* L.) and Larches (*Larix* sp.) and a fourth subspecies, *V. album* subsp. *creticum* N. growing only on Calabrian pine (*Pinus brutia* Ten.)^8,12,13^.

*V. album* is an important medicinal species. Its pharmacological effects such as anti-cancer^14,15^, anti-diabetes, antioxidant^16^, blood pressure-lowering^17^, sedative^18^, antibacterial^19^, antiviral^20^, pro-apoptotic^21^, immunomodulatory^22^ and cytotoxic effects^23^ have been measured through many studies.

The use of herbal drugs obtained from *V. album* in the frame of integrative medicine has been proved to be efficient by reducing chemotherapy induced side effects, increasing quality of life and survival^24–26^. The biological activities of mistletoe are attributed to a wide range of bioactive compounds such as lectins, viscotoxins, flavonoids, phenolic acids, sterols, lignans, terpenoids, phenylpropanoids, alkaloids and fatty acids^27^.

Viscotoxins and mistletoe lectins (ML) are two groups of toxic proteins. Necrosis is the primary effect of viscotoxins whereas mistletoe lectins induce the apoptotic cell death. Both groups have been shown to exert immunomodulatory effects. Therefore it is expected these toxic proteins play an essential role in the treatment of cancer ^9,28,29^.

Mistletoe lectins are sugar-binding proteins, categorized into three types according to their sugar specificity: galactose-specific ML I (115 kDa, dimer), galactose- and N-acetyl-D-galactosamine-specific ML II (60 kDa) and N-acetyl-D-galactosamine-specific ML III (60 kDa)^30–32^.

Viscotoxins are cysteine-rich proteins composed of 46 amino acids and three disulfide bridges. Seven isoforms of viscotoxins have been identified in *V. album*: viscotoxin A1, A2, A3, B, B2, C1 and 1-PS^29,33–35^.

Hyrcanian forests of Iran with a main area of about 1.9 million hectares in northern Iran are located on the northern slopes of the Alborz Mountains and the southern shores of the Caspian Sea. These forests belong to the oldest and most valuable forests of the world protected as an UNESCO world heritage site since 2019^36,37^ Two deciduous trees, Persian ironwood (*Parrotia persica* C.A. Meyer, Hamamelidaceae) and Hornbeam (*Carpinus betulus* L., Betulaceae) are the most frequently colonized host species of *V. album* in the Hyrcanian forests of Iran^38–40^. *Parrotia persica* is an endemic species in these forests^39^.

Previous studies have shown that the quantity and quality of mistletoe bioactive compounds depend on various parameters like ecological characteristics of host species, mistletoe life cycle, and also organ type, which consequently impress the therapeutic properties of mistletoe^10,41–43^. For instance, total phenolic content and antioxidant activity between leaves and stems of *V. album* were different and also were influenced by different host trees and seasons^10^. Viscotoxin and lectin concentrations in leaves of *V. album* were also impressed by seasonal changes^44^. Similarly, the effect of host trees, harvesting time, and organ type on quantity of triterpene acids of *V. album* has been confirmed by other studies^42,45^ To our knowledge, there is no information on viscotoxins and lectins content of *V. album* on *P. persica* and *C. betulus* grown in Hyrcanian forests of Iran. Therefore, this study aimed to evaluate the quantity and quality of viscotoxins and total lectins in stems with leaves (foliage) and fruit of *V. album* on these two Iranian host species in two fruit ripening stages of mistletoe, green berries in September and white berries in December.

## Results

### Total amount of viscotoxins and lectins in foliage and fruit of *V. album* on *P. persica* and *C. betulus* in September and December

**Figure 1:**
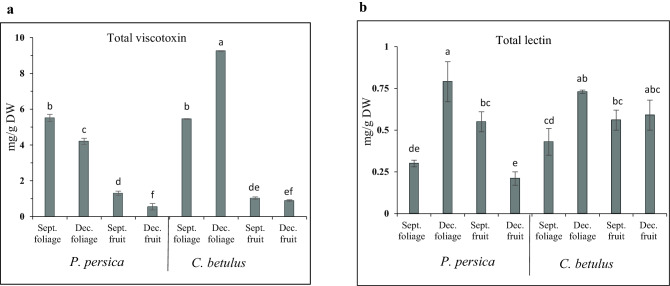
Viscotoxin and lectin concentrations in the foliage and fruit of *V. album* settled on *P. persica* and *C. betulus* in September (Sept.) and December (Dec.). (**a**) Viscotoxins, (**b**) lectins. Each value represents the average of three replicates ± SE. Different letters in figures a and b indicate significant differences at P ≤ 0.01 and P ≤ 0.05 respectively, using the least significant difference test (LSD test).

The analysis of variance indicated that the interaction of host species and mistletoe organ type had a significant effect on viscotoxin and lectin contents at the 1 and 5% probability levels, respectively (Table 1). As shown in Fig. 1a,b, viscotoxins were the prevailing proteins in all samples studied. The highest level of viscotoxins was detected in the December foliage of *V. album* settled on *C. betulus* (9.25 mg/g dry weight [DW]), and the lowest amount was measured in the December fruit of *V. album* from *P. persica* (0.55 mg/g DW). However, the maximum content of viscotoxins in *V. album* from *P. persica* was found in the September foliage (5.51 mg/g DW), which was almost half the maximum amount of viscotoxins in *V. album* from *C. betulus* (Fig. 1a).

**Table 1:** Analysis of variance of viscotoxins and lectins concentrations in the foliage and fruit of *V. album* settled on *P. persica* and *C. betulus* in September and December.

Sorces of variation	DF	Mean square	
		Viscotoxin	Lectin
Host	1	9.60**	0.08*
Organ	3	54.94**	0.18**
Host× organ	3	9.67**	0.6*
Error	23	0.05	0.01
CV		6.08	23.21

*,**Indicate a significant difference at 5% and 1% level, respectively.

It is noticeable that in *V. album* from both host species, the immature green berries in September contained a higher concentration of viscotoxins than the mature white berries in December. The viscotoxin content in the foliage of *V. album* from *P. persica* and *C. betulus* was almost four- and ninefold more concentrated than in fruits, respectively (Fig. 1a).

Regarding the lectins, as depicted in Fig. 1b, the highest concentration was found in the December foliage of *V. album* settled on *P. persic*a (0.79 mg/g DW) and *C. betulus* (0.73 mg/g DW), respectively. The lowest amount of lectins was measured in the December fruit of *V. album* from *P. persica* (0.21 mg/g DW) (Fig. 1b). It is interesting that the green fruit of *V. album* on *P. persica* in September contained about 2- and 2.5-fold higher amounts of lectins (0.55 mg/g DW) than the foliage at the same time (0.3 mg/g DW), and the fruit in December (0.21 mg/g DW), respectively (Fig. 1b). Also, in *V. album* from *C. betulus* in September the lectins content of the green fruit was a little higher (0.56 mg/g DW) in comparison to the September foliage (0.43 mg/g DW) (Fig. 1b).

### Concentrations and portions of viscotoxin isoforms in foliage and fruit of *V. album* grown on *P. persica* and *C. betulus* in September and December

Four isoforms of viscotoxin, viscotoxin A1, A2, A3 and B, could be measured in all samples of *V. album* on both host species. Viscotoxin A3 was the predominant isoform in all samples followed by A1, while A2 had the lowest percentage in all conditions (Table 2).

**Table 2:** Viscotoxin isoforms in the foliage and fruit of *V. album* settled on *P. persica* and *C. betulus* in September and December (% of total viscotoxins).

Host trees	Different organs of *V. album*	Isoforms% of total viscotoxin			
		A3%	A1%	B%	A2%
*P. persica*	September foliage	83.76 ± 0.69^c^	12.6 ± 0.86^e^	1.76 ± 0.09^cd^	1.74 ± 0.11^d^
	December foliage	86.9 ± 0.97^bc^	10.43 ± 0.13^f^	1.46 ± 0.12^cd^	1.17 ± 0.11^de^
	September fruit	73.33 ± 1.1^d^	16.3 ± 0.44^c^	6.7 ± 0.15^b^	3.68 ± 0.17^a^
	December fruit	74.4 ± 0.23^d^	14.99 ± 0.39^d^	8.83 ± 0.38^a^	1.84 ± 0.04^cd^
*C. betulus*	September foliage	87.8 ± 0.55^b^	8.9 ± 0.29^g^	2.4 ± 0.81^c^	0.8 ± 0.15^e^
	December foliage	93 ± 0.26^a^	5.57 ± 0.23^h^	0.93 ± 0.12^d^	0.53 ± 0.12^e^
	September fruit	64.23 ± 2.31^e^	25.37 ± 0.55^a^	7.67 ± 0.18^b^	2.55 ± 0.15^bc^
	December fruit	67.4 ± 1.17^e^	22.93 ± 0.15^b^	6.9 ± 0.40^b^	2.97 ± 0.58^ab^

Each value is the mean ± SE (n= 3). Different letters denote significant differences at P ≤ 0.01 using the least significant difference test (LSD test).

The maximum percentage of A3 (93%) and A1 (25.37%) was detected in the December foliage and September fruit of mistletoe from *C. betulus*, respectively. The maximum percentage of isoforms B and A2 was detected in the December fruit (8.83%) and September fruit (3.68%) of mistletoe from *P. persica*, respectively (Table 2).

The analysis of variance showed that the concentrations (mg/g DW) of viscotoxin isoforms A1, A2, A3 and B were affected by the interaction of host species and organ type at a 1% probability level.

**Figure 2:**
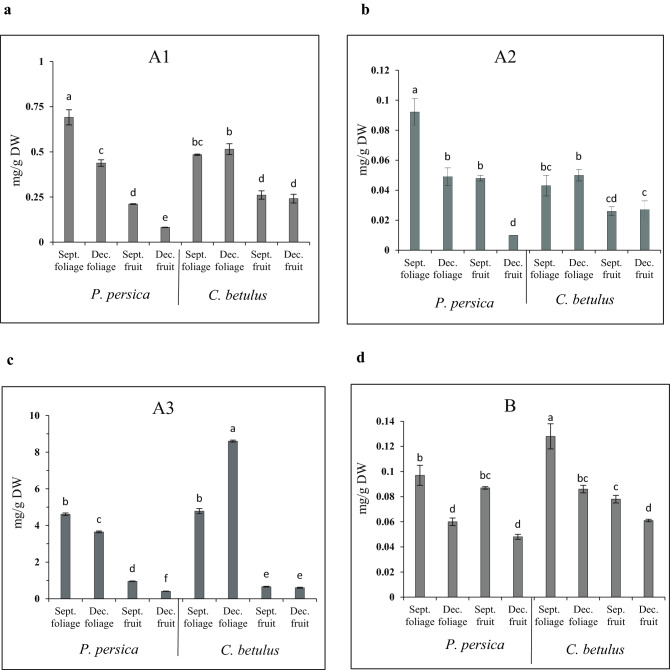
Viscotoxin isoforms concentrations in the foliage and fruit of *V. album* settled on *P. persica* and *C. betulus* at two harvest times. (**a**) Viscotoxin A1, (**b**) Viscotoxin A2, (**c**) Viscotoxin A3 and (**d**) Viscotoxin B. Each value represents the average of three replicates ± SE. Different letters indicate significant differences at P ≤ 0.01 using the least significant difference test (LSD test).

The maximum concentration of viscotoxin A1 (0.69 mg/g DW) and A2 (0.09 mg/g DW) was detected in the September foliage of *V. album* L. from *P. persica* (Fig. 2a,b). The highest concentration of A3 (8.60 mg/g DW) and B (0.13 mg/g DW) was detected in the December and September foliage of mistletoe samples from *C. betulus*, respectively (Fig. 2c,d).

## Discussion

Mistletoe is a hemiparasitic plant that depends on nutrient transfer from the host. Therefore host species might affect phytochemical characteristics and biological activities of mistletoe^10^. It has been shown that also harvest time and type of organ produce variations in the chemical composition of mistletoe^29^. In this study two groups of the most important bioactive compounds, viscotoxins and lectins, have been analyzed in *V. album* grown on *P. persica* and *C. betulus* for the first time. Total viscotoxin, its isoforms and mistletoe lectin were quantified in the foliage and fruit of *V. album* in the phenological stages with green berries in September and white berries in December.

Our findings showed significant differences in the quantity and quality of viscotoxins and total lectins depending on host tree, season, and organ type of mistletoe. The highest level of viscotoxins and lectins in *V. album* from *P. persic*a was achieved in September and December respectively and in case of samples from *C. betulus*, both compounds were in the highest concentrations in December. Seasonal and host dependent variations of different bioactive compounds in mistletoe have already been found in *V. album* (different compounds^41^, antioxidants^46,47^, viscotoxins and lectins^44^). Consistent with the present results, Urech et al.^44^ detected the maximum concentration of lectins and viscotoxins in *V. album* subsp. *album* leaves in December and June, respectively.

Our results regarding the effect of mistletoe organ type on viscotoxin and lectin accumulation showed that the mistletoe foliage contained higher amounts of these compounds in comparison with the fruit. Such a preferential accumulation of compounds in the leaves of *V. album* has been shown before also for other compounds. Soursouri et al.^45^ reported that the highest amount of triterpene acids was detected in the foliage, and Stefanucci et al.^43^ measured the highest levels of total phenolics, flavonoids, and antioxidant effect of *V*. *album* L. in leaf extract compared with fruit and seed.

The highest concentrations of ML in this study extracted from the last three generations of *V. album* foliage in December (0.73–0.8 mg/g DW) were within the range of ML concentrations related to the first (1.8 mg/g DW) and second (0.4 mg/g DW) year of growth of *V. album* subsp. *album* leaves reported by Urech et al.^44^.

The concentrations of total viscotoxins detected here (4.20–9.25 mg/g DW in the foliage of *V. album*) corresponded almost to the concentrations evaluated in *V. album* subsp. *album* on different deciduous host trees by Schaller et al.^48^ and Holandino et al.^49^ who measured total viscotoxins between 2.2 and 5.7 mg/g fresh weight (FW) and 2.42–3.95 mg/g FW respectively.

The isoforms viscotoxin A1, A2, A3, and B known to be present in *V. album* could be assessed in all samples of *V. album* measured here. However, the composition of the viscotoxin isoforms in the mistletoe samples from the two Iranian deciduous host trees *P. persica* and *C. betulus* did not correspond to the composition in the European *V. album* subsp. *album*. The extremely high portion of about 87% and 93% viscotoxin A3 and the low concentrations of viscotoxin A2 comply with the viscotoxin pattern of *V. album* subsp. *abietis* growing exclusively on fir (*Abies* ssp.)^44^. The European *V. album* subsp. *album* growing on deciduous trees contains rather well-balanced portions of the four viscotoxin isoforms^44^ so that viscotoxin A2 and A3 are present in almost equivalent portions in this subspecies. Previous studies on *V. album* subsp. *album* from different host species have shown that the maximum percentage of each viscotoxin isoform in this subspecies was A1: 16%, A2: 35%, A3: 41.2% and B: 14%^44^ meanwhile in *V. album* subsp. *abietis* these percentages changed to A1: 6.5%, A2: not detected, A3: 74.5% and B: 5.4%^44^ being close to the portions measured in our samples of *C. betulus* and *P. persica*. This correspondence leads us to the hypothesis that the Iranian *V. album* on the two host species might represent a specific genetic variant of *V. album*. Impact of ecological factors on the composition of these small proteins is very unlikely in view of the constant viscotoxin patterns detected in *V. album* subsp. *album* on 7 different host species representing a big range of ecological conditions^29,48^.

Zuber and Widmer^50^ concluded in their study on genetic and geographic differentiation of *V. album*, that highly differentiated populations and possibly new taxa exist at the range limit of this species. Iranian mistletoe belongs to one of the easternmost populations of *V. album.*

Our study detected significant variations in quantity and quality of viscotoxins and total lectins of *V. album* under the effect of the host species and mistletoe organ type at two stages of fruit development in September with green berries and in December with white berries. It has to be expected that the pharmacological activities of mistletoe extracts such as cytotoxicity and induction of apoptosis in cancer cells vary accordingly. It has been shown that the four isoforms, viscotoxin A1, A2, A3, and B, have cytotoxic effects on cancer cell lines. Viscotoxin A3 proved to be the isoform with the highest specific activity^49,51,52^. Therefore it is expected that the Iranian *V. album* on the two host species measured with high concentrations of viscotoxin A3 exert high viscotoxin dependent cytotoxic effects exceeding that of the European *V*. *album* subsp. *album*. It will be crucial for the pharmaceutical use of *V. album* to take into consideration the results presented here.

## Materials and methods

### Plant material

The collection of plant samples in this study was done according to legislation and formal permission of Iran Natural Resources and Watershed Management organization. *Viscum album* L. plants were harvested in 2019 from two host species, *P. persica* C.A. Meyer and *C. betulus* L., located in the kelerd forest of the Hyrcanian forests in Mazandaran province of Iran (Table 3) in September (mistletoe green berry stage) and December (mistletoe white berry stage). The altitude of this region is between 700 and 1100 m above sea level, and the average annual rainfall is 668 mm.

**Table 3:** Host species of *V. album* and their location.

Host species	Family	Study area	Coordinates	
			Latitude	Longitude
*Carpinus betulus L.*	Betulaceae	Kelerd forest of the Hyrcanian forests, Iran	36° 16′ 3′′	52° 21′ 7′′
*Parrotia persica* C. A. Mey.	Hamamelidaceae	Kelerd forest of the Hyrcanian forests, Iran	36° 16′ 1′′	52° 21′ 13′′

**Figure 3:**
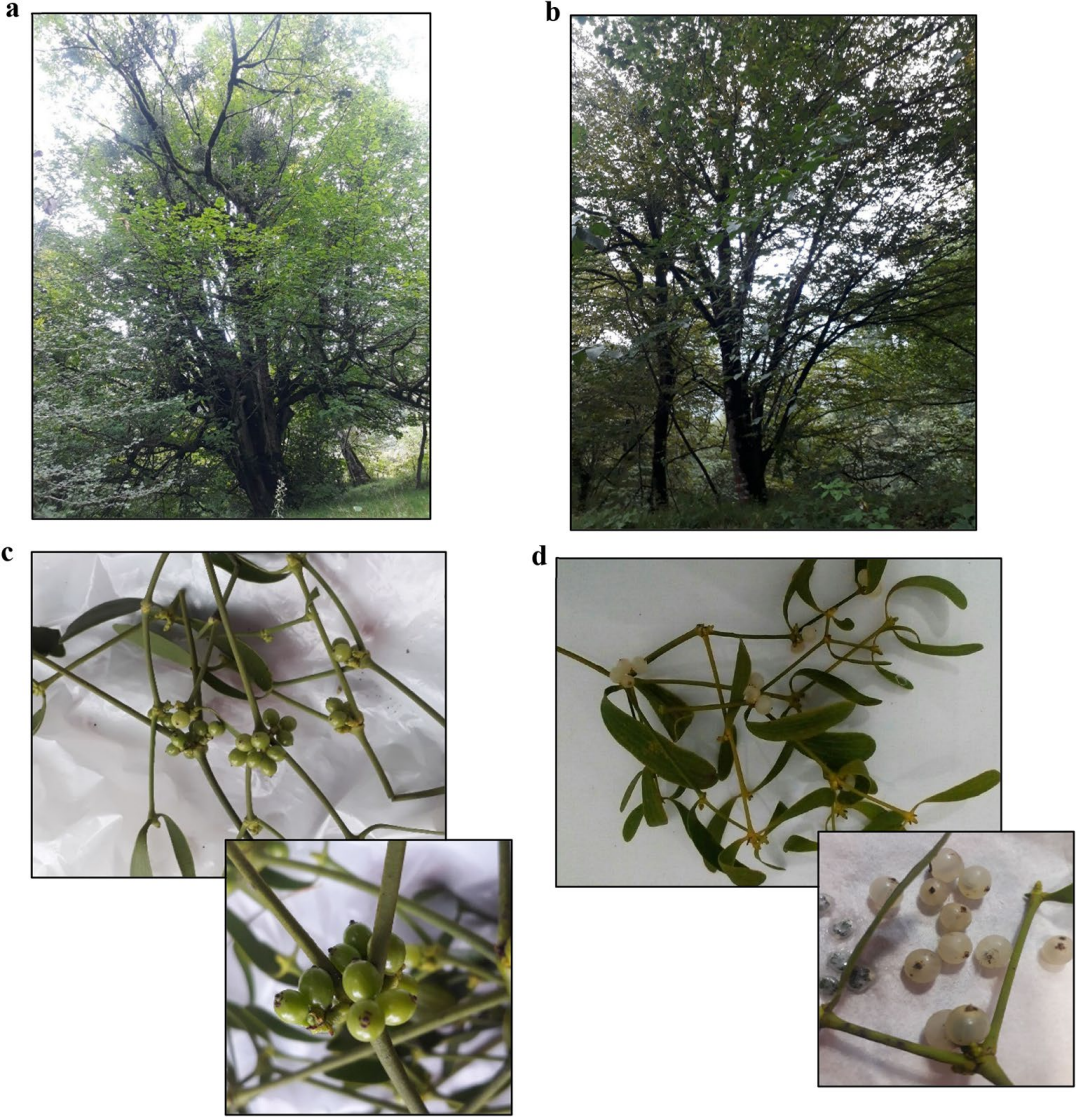
Sampling of mistletoe plants from 2 different host trees. (**a**) *P. persica*, (**b**) *C. betulus*, (**c**) mistletoe in green berry phenological stage and (**d**) mistletoe in white berry phenological stage.

For sampling, three individual trees of each host species which were similar in diameter, height and morphology were selected and three mistletoe plants were marked on each of these trees in order to perform harvesting in September and December on the same mistletoe plants. In both harvesting times five branches of each mistletoe plant including the last three generations of organs (foliage and fruit) were collected (Fig. 3), placed in liquid nitrogen for a few seconds and finally stored at -80 °C until extraction. In the following the designation “foliage” is used for the mistletoe branch including leaves and stems without fruit.

### Preparation of extracts

The mistletoe foliage and fruit were separately freeze-dried for 72 h. Then the dried samples of foliage or fruits were powdered in liquid nitrogen using mortar and pestle.

#### Viscotoxins extraction

For this extraction, 200 mg of the plant powder was mixed with 5 ml of 2% acetic acid and left overnight at 4 °C in 3 replicates. The resulting mixture was then homogenized by an Ultra-turrax homogenizer. The samples were centrifuged at 4000 × g for 30 min and the supernatant was separated. The extraction process was repeated by adding new solvent to the pellet. The two supernatants were pooled and the volume adjusted to 10 ml. These viscotoxin extracts were stored at 4 °C until HPLC analysis^44^.

#### Lectins extraction

Regarding lectins extraction, 200 mg of the plant powder was suspended in 5 ml of phosphate buffered saline (PBS) and left overnight at 4 °C in 3 replicates. After homogenizing by an Ultra-turrax homogenizer the mixture was centrifuged at 4000 × g for 30 min, and the supernatant was separated. This procedure was repeated with the pellet and 5 ml PBS and once again by adding 5 ml of PBS containing 0.2 M galactose. The total volume of the combined extracts was adjusted to 15 ml and stored at − 20 °C until chemical evaluation^44^.

### Biochemical analysis

#### Viscotoxins

The quantity of viscotoxin isoforms (A1, A2, A3, B) were analyzed with an Agilent 1100 HPLC system equipped with Nucleosil C18 AB, 125 × 4 mm column and UV detector (210 nm). The gradient mobile phase comprised eluent A (0.1% TFA in water) and eluent B (0.1% TFA in acetonitrile/water 60/40) which changed from 38% B to 42% B in 9 min with a subsequent step to 50% B and an increase to 54% B in 8 min with a 1 ml/min flow rate. A standard curve was drawn according to the surface under the curve of four viscotoxin standards^44^.

#### Mistletoe lectins

Total ML was determined by ELISA using monoclonal antiMLA-5F5 (capture antibody) and antiML-B-5H8 (detector antibody) according to the method of Jäggy et al.^44,53^.

### Statistical analysis

The statistical analysis was performed using SAS 9 software. The residual normality of data was tested using the Shapiro–Wilk test. All the experiments were analyzed by two-way ANOVA (analysis of variance), and significant differences between means were compared by the least significant difference test (LSD) at the 0.05 probability level.

## Conclusion

In this study, the quantity and quality of viscotoxins and also total lectins of the foliage and fruit of *V. album* L. plants growing on *P. persica* and *C. betulus* in Hyrcanian forests of Iran were evaluated in September in the phenological state of green berries and in December with white berries for the first time. Our findings indicate significant variations in the concentrations of two groups of pharmacologically important antitumor compounds, viscotoxins and lectins, depending on host species, mistletoe organ type, and harvest time. On the whole, viscotoxins were the prevailing proteins in all studied samples. Among the two mistletoe organs tested, the foliage extract contained the highest amount of both compounds. The foliage of *V. album* on *C. betulus* in December had the highest level of viscotoxins. The maximum amount of lectins was found in the December foliage of *V. album* settled on *P. persica* and *C. betulus*. In the mistletoes from both host species, the green berries in September showed higher contents of viscotoxins and lectins than the white berries in December. Regarding viscotoxin composition, 4 isoforms of viscotoxin, A1, A2, A3, and B, were detected in all samples of both host species. A3 was by far the most predominant viscotoxin isoform followed by A1 in all samples, whereas A2 represented the lowest concentration. This extraordinary pattern of viscotoxins in the Iranian *V. album* on the two deciduous trees contrasts with the patterns known for *V. album* subsp. *album* growing on deciduous trees in Europe. This points to possible new genetic variants of *V. album* in Iran which can have more potent cytotoxic activity than European *V. album* subsp. *album*.

## Data Availability

Data sharing is not applicable to this article as all new created data is already contained within this article.
